# Supplementary material for: Cellular and Humoral Responses to Recombinant and Inactivated SARS-CoV-2 Vaccines in CKD Patients: An Observational Study
Source: J Clin Med. 2023 Feb 3;12(3):1225. doi: 10.3390/jcm12031225 (PMC9918183; doi:10.3390/jcm12031225)
Supplement: Supplementary file 1 [file jcm-12-01225-s001.zip › Supplementary Material S1.pdf]

Snibe Diagnostic

CE REF 130269017M:100 tests/kit
130669017M: 50 tests/kit
130769017M: 30 tests/kit

MAGLUMI® SARS-CoV-2 S-RBD IgG II (CLIA)

INTENDED USE

The kit is an *in vitro* chemiluminescence immunoassay for the quantitative determination of S-RBD IgG antibodies to SARS-CoV-2 in human serum and plasma using the MAGLUMI series Fully-auto chemiluminescence immunoassay analyzer.

SUMMARY

SARS-CoV-2, formerly called 2019-nCoV, is a betacoronavirus and cause an acute respiratory disease (coronavirus disease-2019, COVID-19). Coronavirus is an envelope virus with four structural proteins: spike (S) protein, membrane (M) protein, envelope (E) protein and nucleocapsid (N) protein. Among the 4 coronavirus structural proteins, the S and N proteins are the main immunogens. Specially, S protein is a major protective antigen that elicits highly potent neutralizing antibodies (NAbs) and plays an essential role in viral attachment, fusion, entry, and transmission. S protein comprises a N-terminal S1 subunit responsible for virus-receptor binding and a C terminal S2 subunit responsible for virus-cell membrane fusion. S1 is further divided into a N-terminal domain (NTD) and a receptor-binding domain (RBD), and RBD within S1 subunit directly interacts with host receptors. Human angiotensin coverting enzyme 2 (hACE2) is the receptor to which SARS-CoV-2 binds to enter host cells.

NAbs are antibodies that are capable of keeping an infectious agent (for instance, virus) from infecting a cell by neutralizing or inhibiting its biological effect. RBD within S1 subunit is the most critical target for SARS-CoV NAbs. Such NAbs can interrupt the interaction of RBD and its receptor ACE2. SARS-CoV-2 S-RBD IgG II assay is to be used as an aid in identifying patients with an adaptive immune response to SARS-CoV-2, indicating recent or prior infection in conjunction with clinical presentation and other laboratory tests. Results from the SARS-CoV-2 IgG S-RBD IgG II assay should not be used as the sole basis for diagnosis.

TEST PRINCIPLE

Indirect chemiluminescence immunoassay.

The sample, buffer and magnetic microbeads coated with recombinant SARS-CoV-2 S-RBD antigen are mixed thoroughly and incubated, performing a wash cycle after a precipitation in a magnetic field. ABEI labeled with anti-human IgG antibody are then added and incubated, reacting to form immuno-complexes. After precipitation in a magnetic field, the supernatant is decanted and then a wash cycle is performed. Subsequently, the Starter 1+2 are added to initiate a chemiluminescent reaction. The light signal is measured by a photomultiplier as relative light units (RLUs), which is proportional to the concentration of SARS-CoV-2 S-RBD IgG present in the sample.

REAGENTS

Kit Contents

Component	Description	100 tests/kit	50 tests/kit	30 tests/kit
**Magnetic Microbeads**	Magnetic microbeads coated with recombinant SARS-CoV-2 S-RBD antigen in PBS buffer, NaN_3 (<0.1%).	2.5 mL	1.5 mL	1.0 mL
**Calibrator Low**	Low concentration of SARS-CoV-2 S-RBD IgG antibodies in PBS buffer, NaN_3 (<0.1%).	1.0 mL	1.0 mL	1.0 mL
**Calibrator High**	High concentration of SARS-CoV-2 S-RBD IgG antibodies in PBS buffer, NaN_3 (<0.1%).	1.0 mL	1.0 mL	1.0 mL
**Buffer**	NaCl and BSA, NaN_3 (<0.1%).	22.5 mL	12.0 mL	7.8 mL
**ABEI Label**	ABEI labeled with anti-human IgG antibody in Tris-HCl buffer, NaN_3 (<0.1%).	22.5 mL	12.0 mL	7.8 mL
**Diluent**	PBS buffer, NaN_3 (<0.1%).	5.5 mL	3.5 mL	3.5 mL
**Control 1**	SARS-CoV-2 S-RBD IgG antibodies in PBS buffer, NaN_3 (<0.1%).	1.0 mL	1.0 mL	1.0 mL
**Control 2**	SARS-CoV-2 S-RBD IgG antibodies in PBS buffer, NaN_3 (<0.1%).	1.0 mL	1.0 mL	1.0 mL
**Control 3**	SARS-CoV-2 S-RBD IgG antibodies in PBS buffer, NaN_3 (<0.1%).	1.0 mL	1.0 mL	1.0 mL
All reagents are provided ready-to-use.				

Warnings and Precautions

- For *in vitro* diagnostic use.
- For professional use only.
- Exercise the normal precautions required for handling all laboratory reagents.
- Personal protective measures should be taken to prevent any part of the human body from contacting samples, reagents, and controls, and should comply with local operating requirements for the assay.
- A skillful technique and strict adherence to the package insert are necessary to obtain reliable results.
- Do not use kit beyond the expiration date indicated on the label.
- Do not interchange reagent components from different reagents or lots.
- Avoid foam formation in all reagents and sample types (specimens, calibrators and controls).
- All waste associated with biological samples, biological reagents and disposable materials used for the assay should be considered potentially infectious and should be disposed of in accordance with local guidelines.
- This product contains sodium azide. Sodium azide may react with lead or copper plumbing to form highly explosive metal azides. Immediately after disposal, flush with a large volume of water to prevent azide build-up. For additional information, see Safety Data Sheets available for professional user on request.

Note: If any serious incident has occurred in relation to the device, please report to Shenzhen New Industries Biomedical Engineering Co., Ltd. (Snibe) or our authorized representative and the competent authority of the Member State in which you are established.

Reagent Handling

- To avoid contamination, wear clean gloves when operating with a reagent kit and sample. When handling reagent kit, replace the gloves that have been in contact with samples, since introduction of samples will result in unreliable results.
- Do not use kit in malfunction conditions; e.g., the kit leaking at the sealing film or elsewhere, obviously turbid or precipitation is found in reagents (except for Magnetic Microbeads) or control value is out of the specified range repeatedly. When kit in malfunction conditions, please contact Snibe or our authorized distributor.
- To avoid evaporation of the liquid in the opened reagent kits in refrigerator, it is recommended that the opened reagent kits to be sealed with reagent seals contained within the packaging. The reagent seals are single use, and if more seals are needed, please contact Snibe or our authorized distributor.
- Over time, residual liquids may dry on the septum surface. These are typically dried salts and have no effect on assay efficacy.
- Use always the same analyzer for an opened reagent integral.
- For magnetic microbeads mixing instructions, refer to the Preparation of the Reagent section of this package insert.
- For further information about the reagent handing during system operation, please refer to Analyzer Operating Instructions.

Storage and Stability

- Do not freeze the integral reagents.
- Store the reagent kit upright to ensure complete availability of the magnetic microbeads.
- Protect from direct sunlight.

**Stability of the Reagents**	
Unopened at 2-8°C	until the stated expiration date
Opened at 2-8°C	6 weeks
On-board	4 weeks

Stability of Controls	
Unopened at 2-8°C	until the stated expiration date
Opened at 2-8°C	6 weeks
Frozen at -20°C	2 months
Frozen and thawed cycles	no more than 2 times

SPECIMEN COLLECTION AND PREPARATION

Specimen Types

Only the specimens listed below were tested and found acceptable.

Specimen Types	Collection Tubes
**Serum**	Tubes without additive / accessory, or tubes containing clot activator or clot activator with gel.
**Plasma**	K2-EDTA, K3-EDTA or Na2-EDTA

- The sample types listed were tested with a selection of sample collection tubes that were commercially available at the time of testing, i.e. not all available tubes of all manufacturers were tested. Sample collection systems from various manufacturers may contain differing materials which could affect the test results in some cases. Follow tube manufacturers' instructions carefully when using collection tubes.

Specimen Conditions

- Do not use grossly hemolyzed/hyperlipidaemia specimens and specimens with obvious microbial contamination.
- Ensure that complete clot formation in serum specimens has taken place prior to centrifugation. Some serum specimens, especially those from patients receiving anticoagulant or thrombolytic therapy, may exhibit increased clotting time. If the serum specimen is centrifuged before a complete clotting, the presence of fibrin may cause erroneous results.
- Samples must be free of fibrin and other particulate matter.
- To prevent cross contamination, use of disposable pipettes or pipette tips is recommended.

Preparation for Analysis

- Inspect all specimens for foam. Remove foam with an applicator stick before analysis. Use a new applicator stick for each specimen to prevent cross contamination.
- Frozen specimens must be completely thawed before mixing. Mix thawed specimens thoroughly by low speed vortexing or by gently inverting. Visually inspect the specimens. If layering or stratification is observed, mix until specimens are visibly homogeneous. If specimens are not mixed thoroughly, inconsistent results may be obtained.
- Specimens should be free of fibrin, red blood cells, or other particulate matter. Such specimens may give reliable results and must be centrifuged prior to testing. Transfer clarified specimen to a sample cup or secondary tube for testing. For centrifuged specimens with a lipid layer, transfer only the clarified specimen and not the lipemic material.
- The sample volume required for a single determination of this assay is 10 µL.

Specimen Storage

Specimens removed from the separator, red blood cells or clot may be stored up to or 3 days at 10-30°C, or 7 days at 2-8°C, or 2 months frozen at -20°C. Frozen specimens subjected to up to 2 freeze/thaw cycles have been evaluated.

Specimen Shipping

- Package and label specimens in compliance with applicable local regulations covering the transport of clinical specimens and infectious substances.
- Do not exceed the storage limitations listed above.

Specimen Dilution

- Samples, with SARS-CoV-2 S-RBD IgG II concentrations above the analytical measuring interval, can be diluted with Diluent either by following automated dilution protocol or manual dilution procedure. The recommended dilution ratio is 1:4. The concentration of the diluted sample must be >200 AU/mL.
- For manual dilution, multiply the result by the dilution factor. For dilution by the analyzers, the analyzer software automatically takes the dilution into account when calculating the sample concentration.

PROCEDURE

Materials Provided

SARS-CoV-2 S-RBD IgG II (CLIA) assay, control barcode labels.

Materials Required (But Not Provided)

- General laboratory equipment.
- Fully-auto chemiluminescence immunoassay analyzer Maglumi 600, Maglumi 800, Maglumi 1000, Maglumi 2000, Maglumi 2000 Plus, Maglumi 4000, Maglumi 4000 Plus, MAGLUMI X8, MAGLUMI X3.
- Additional accessories of test required for the above analyzers include Reaction Module, Starter 1+2, Wash Concentrate, Light Check, Tip, and Reaction Cup. Specific accessories and accessories' specification for each model refer to corresponding Analyzer Operating Instructions.
- Please use accessories specified by Snibe to ensure the reliability of the test results.

Assay Procedure

Preparation of the Reagent

- Take the reagent kit out of the box and visually inspect the integral vials for leaking at the sealing film or elsewhere. If there is no leakage, please tear off the sealing film carefully.
- Open the reagent area door; hold the reagent handle to get the RFID label close to the RFID reader (for about 2s); the buzzer will beep; one beep sound indicates successful sensing.
- Keeping the reagent straight insert to the bottom along the blank reagent track.
- Observe whether the reagent information is displayed successfully in the software interface, otherwise repeat the above two steps.
- Resuspension of the magnetic microbeads takes place automatically when the kit is loaded successfully, ensuring the magnetic microbeads are totally resuspended homogenous prior to use.

Assay Calibration

- Select the assay to be calibrated and execute calibration operation in reagent area interface. For specific information on ordering calibrations, refer to the calibration section of Analyzer Operating Instructions.
- Execute recalibration according to the calibration interval required in this package insert.

Quality Control

- When new lot used, check or edit the quality control information.
- Scan the control barcode, choose corresponding quality control information and execute testing. For specific information on ordering quality controls, refer to the quality control section of the Analyzer Operating Instructions.

Sample Testing

- After successfully loading the sample, select the sample in interface and edit the assay for the sample to be tested and execute testing. For specific information on ordering patient specimens, refer to the sample ordering section of the Analyzer Operating Instructions.

To ensure proper test performance, strictly adhere to Analyzer Operating Instructions.

Calibration

Traceability: This method has been standardized against the Snibe internal reference standard.

Test of assay specific calibrators allows the detected relative light unit (RLU) values to adjust the master curve.

Recalibration is recommended as follows:

- Whenever a new lot of Reagent or Starter 1+2 is used.
- Every 14 days.
- The analyzer has been serviced.
- Control values lie outside the specified range.

Quality Control

Controls are recommended for the determination of quality control requirements for this assay and should be run in singlicate to monitor the assay performance. Refer to published guidelines for general quality control recommendations, for example Clinical and Laboratory Standards Institute (CLSI) Guideline C24 or other published guidelines[1].

Quality control is recommended once per day of use, or in accordance with local regulations or accreditation requirements and your laboratory's quality control procedures, quality control could be performed by running the SARS-CoV-2 S-RBD IgG II assay:

- Whenever the kit is calibrated.
- Whenever a new lot of Starter 1+2 or Wash Concentrate is used.

Controls are only applicable with MAGLUMI and only used matching with the same top seven LOT numbers of corresponding reagents. For each target value and range refer to the label.

The performance of other controls should be evaluated for compatibility with this assay before they are used. Appropriate value ranges should be established for all quality control materials used.

Control values must lie within the specified range, whenever one of the controls lies outside the specified range, calibration should be repeated and controls retested. If control values lie repeatedly outside the predefined ranges after successful calibration, patient results must not be reported and take the following actions:

- Verify that the materials are not expired.
- Verify that required maintenance was performed.
- Verify that the assay was performed according to the package insert.
- If necessary, contact Snibe or our authorized distributors for assistance.

RESULTS

Calculation

The analyzer automatically calculates the SARS-CoV-2 S-RBD IgG II concentration in each sample by means of a calibration curve which is generated by a 2-point calibration master curve procedure. The results are expressed in AU/mL. For further information please refer to the Analyzer Operating Instructions.

Interpretation of Results

Results of reference range study using the SARS-CoV-2 S-RBD IgG II assay was as follows:

Non-reactive: A result less than 1.00 AU/mL (<1.00 AU/mL) is considered to be non-reactive.

Reactive: A result greater than or equal to 1.00 AU/mL (≥1.00 AU/mL) is considered to be reactive.

For samples with concentration near the cut-off or positive, follow-up tests should be performed.

Results may differ between laboratories due to variations in population and test method. It is recommended that each laboratory establish its own reference interval.

LIMITATIONS

- This test is suitable only for investigating single samples, not for pooled samples.
- Results should be used in conjunction with patient's medical history, clinical examination and other findings.
- Assay results should not be used as the sole basis for the diagnosis and exclusion of novel coronavirus pneumonia, but only as a supplement to existing viral nucleic acid detection reagents and imaging features.
- If the SARS-CoV-2 S-RBD IgG II results are inconsistent with clinical evidence, additional testing is needed to confirm the result.
- Specimens from patients who have received preparations of mouse monoclonal antibodies for diagnosis or therapy may contain human anti-mouse antibodies (HAMA). Such specimens may show either falsely elevated or depressed values when tested with assay kits which employ mouse monoclonal antibodies[2,3]. Additional information may be required for diagnosis.
- Long-term frozen samples may be partially coagulated, which could lead slightly higher results (mainly 1.00 AU/mL-2.00 AU/mL). If a positive result was get when measuring frozen samples, repeated test should be conducted after centrifuged especial severely or using additional test to confirm the result.
- Heterophilic antibodies in human serum can react with reagent immunoglobulins, interfering with *in vitro* immunoassays. Patients routinely exposed to animals or animal serum products can be prone to this interference and anomalous values may be observed[4].
- Bacterial contamination of the specimens may affect the test results.

SPECIFIC PERFORMANCE CHARACTERISTICS

Representative performance data are provided in this section. Results obtained in individual laboratories may vary.

Precision

Precision was determined using the assay, samples and controls in a protocol (EP05-A3) of the CLSI (Clinical and Laboratory Standards Institute): duplicates at two independent runs per day for 5 days at three different sites using three lots of reagent kits (n = 180). The following results were obtained:

Sample	Mean (AU/mL)	N	Repeatability		Between-Lot		Between-Day		Between-Site		Reproducibility	
			SD (AU/mL)	%CV	SD (AU/mL)	%CV	SD (AU/mL)	%CV	SD (AU/mL)	%CV	SD (AU/mL)	%CV
Serum 1	1.092	180	0.051	4.67	0.037	3.39	0.016	1.47	0.076	6.96	0.080	7.33
Serum 2	2.013	180	0.084	4.17	0.028	1.39	0.020	0.99	0.104	5.17	0.116	5.76
Serum 3	951.843	180	4.912	0.52	1.642	0.17	3.638	0.38	6.606	0.69	14.028	1.47
Plasma 1	1.150	180	0.052	4.52	0.047	4.09	0.036	3.13	0.087	7.57	0.087	7.57
Plasma 2	2.153	180	0.081	3.76	0.029	1.35	0.065	3.02	0.121	5.62	0.131	6.08
Plasma 3	960.765	180	4.951	0.52	1.688	0.18	3.614	0.38	6.613	0.69	11.524	1.20
Control 1	0.404	180	0.043	N/A	0.012	N/A	0.025	N/A	0.055	N/A	0.064	N/A
Control 2	4.007	180	0.046	1.15	0.007	0.17	0.022	0.55	0.052	1.30	0.110	2.75
Control 3	14.879	180	0.159	1.07	0.093	0.63	0.096	0.65	0.223	1.50	0.262	1.76

N/A = Not applicable

Linear Range

0.375-1000 AU/mL (defined by the Limit of Quantitation and the maximum of the master curve).

Reportable Interval

0.200-5000 AU/mL (defined by the Limit of Detection and the maximum of the master curve × Recommended Dilution Ratio).

Analytical Sensitivity

Limit of Blank (LoB) =0.100 AU/mL.

Limit of Detection (LoD) =0.200 AU/mL.

Limit of Quantitation (LoQ) =0.375 AU/mL.

Analytical Specificity

Interference

Interference was determined using the assay, two samples containing different concentrations of analyte were spiked with potential endogenous and exogenous interferents in a protocol (EP7-A2) of the CLSI. The measurement deviation of the interference substance is within ±10%. The following results were obtained:

Interference	No interference up to	Interference	No interference up to	Interference	No interference up to
Bilirubin	40 mg/dL	Levofloxacin	1.776 mg/dL	Mometasone	2.5 mg/dL
Intralipid	1000 mg/dL	Azithromycin	1.201 mg/dL	Budesonide	3.2 mg/dL
Hemoglobin	2000 mg/dL	Ribavirin	90 mg/dL	Mucin	260 mg/dL
HAMA	30 ng/mL	Meropenem	80.15 mg/dL	Zanamivir	1.2 mg/dL
Rheumatoid Factor	1500 IU/mL	Tobramycin	2.4 mg/dL	Peramivir	60 mg/dL

ANA	400 AU/mL	Oseltamivir	1.0 mg/dL	Lopinavir	48 mg/dL
Anti-Mitochondrial	1:64 (titer)	Oxymetazoline	2.5 mg/dL	Ritonavir	120 mg/dL
Total IgG	1600 mg/dL	Sodium chloride	45 mg/dL	Arbidol	36 mg/dL
Total IgM	280 mg/dL	Beclomethasone	2.5 mg/dL	Flunisolide	2.5 mg/dL
Interferon α	1500 U/mL	Dexamethasone	18 mg/dL	Histamine dihydrochloride	4.5 mg/dL
Phenylephrine hydrochloride	1.0 mg/dL	Triamcinolone acetonide	5.5 mg/dL	Biotin	5.0 mg/dL
Fluticasone propionate	2.5 mg/dL	Ceftriaxone sodium	81.03 mg/dL	-	-

Cross-Reactivity

The cross-reactivity study for the SARS-CoV-2 S-RBD IgG II (CLIA) assay was designed to evaluate potential cross reactants. The results are listed in the following table:

Category	N of samples	Reactive	Category	N of samples	Reactive
Human Coronavirus antibodies (HKU1, OC43, NL63, 229E)	30	0	Measles virus antibodies	9	0
Influenza A virus antibodies	32	0	CMV antibodies	11	0
Influenza B virus antibodies	14	0	Rotavirus antibodies	7	0
Respiratory syncytial virus antibodies	7	0	Norovirus antibodies	6	0
Rhinovirus antibodies	20	0	Mumps virus antibodies	8	0
Adenovirus antibodies	51	0	Varicella zoster virus antibodies	7	0
Enterovirus antibodies	29	0	M.Pneumoniae antibodies	8	0
EB virus antibodies	15	0	Human immunodeficiency virus	17	0

High-Dose Hook

No high-dose hook effect was seen for SARS-CoV-2 S-RBD IgG II concentrations up to 7000 AU/mL.

Method Comparison

A comparison of the SARS-CoV-2 S-RBD IgG II assay with a commercially available immunoassay, gave the following correlations (AU/mL):

Number of samples measured: 203

Passing-Bablok: y=1.0008x+0.0075, τ=0.984.

The clinical specimen concentrations were between 0.519 and 941.6 AU/mL.

Clinical Sensitivity

The clinical sensitivity of the SARS-CoV-2 S-RBD IgG II assay was determined by testing 212 samples confirmed COVID-19 infected specimens.

Days Post Onset of Symptoms	N of samples	Reactive	Sensitivity	95% CI
0-7	59	37	62.71%	49.95%-73.92%
8-14	75	69	92.00%	83.63%-96.28%
≥ 15	78	78	100.00%	95.31%-100.00%

The positive rate of S-RBD IgG antibodies may be affected by the infection period of the test subject (when blood sampling) in different studies.

Clinical Specificity

The clinical specificity of the SARS-CoV-2 S-RBD IgG II assay was determined by testing 327 samples from subjects assumed to be negative for SARS-CoV-2.

N of samples	Non-reactive	Specificity	95% CI
327	326	99.69%	98.29%-99.95%

REFERENCES

1. CLSI. Statistical Quality Control for Quantitative Measurement Procedures: Principles and Definitions. 4th ed. CLSI guideline C24. Wayne, PA: Clinical and Laboratory Standards Institute; 2016.
2. Schroff R W, Foon K A, Beatty S M, et al. Human anti-murine immunoglobulin responses in patients receiving monoclonal antibody therapy. Cancer Research, 1985, 45(2): 879-885.
3. Primus F J, Kelley E A, Hansen H J, et al. "Sandwich"-type immunoassay of carcinoembryonic antigen in patients receiving murine monoclonal antibodies for diagnosis and therapy. Clinical Chemistry, 1988, 34(2): 261-264.
4. Boscato L M, Stuart M C. Heterophilic antibodies: a problem for all immunoassays [J]. Clinical Chemistry, 1988, 34(1): 27-33.

SYMBOLS EXPLANATIONS

	Consult instructions for use		Manufacturer
2 °C 8 °C	Temperature limit (Store at 2-8°C)		Use-by date
Σ	Contains sufficient for<n> tests		Keep away from sunlight
	This way up	EC REP	Authorized representative in the European Community
IVD	*In vitro* diagnostic medical device	CONTENTS	Kit component
REF	Catalogue number	LOT	Batch code
CE	CE marking		

MAGLUMI® is trademark of Snibe. All other product names and trademarks are the property of their respective owners.

Shenzhen New Industries Biomedical Engineering Co., Ltd.
No.23, Jinxiu East Road, Pingshan District, 518122 Shenzhen, P.R. China
Tel: +86-755-21536601 Fax:+86-755-28292740

EC REP
Shanghai International Holding Corp. GmbH (Europe)
Eiffestrasse 80, 20537 Hamburg, Germany
Tel: +49-40-2513175 Fax: +49-40-255726

MAGLUMI® SARS-CoV-2 Neutralizing Antibody (CLIA)

INTENDED USE

The kit is an *in vitro* chemiluminescence immunoassay for the quantitative determination of neutralizing antibodies to SARS-CoV-2 in human serum and plasma using the MAGLUMI series Fully-auto chemiluminescence immunoassay analyzer, and the assay is used for an aid in identifying individuals with an adaptive immune response to SARS-CoV-2, indicating recent or prior infection. The kit should not be used to diagnose acute SARS-CoV-2 infection.

SUMMARY

SARS-CoV-2, formerly called 2019-nCoV, is an enveloped non-segmented positive-sense RNA virus and cause an acute respiratory disease (coronavirus disease-2019, COVID-19). SARS-CoV-2 has several structural proteins including spike (S), envelope (E), membrane (M) and nucleocapsid (N). Among the proteins, the spike (S) and nucleocapsid (N) proteins are the main immunogens. Specially, S protein is a major protective antigen that elicits highly potent neutralizing antibodies (NAbs) and plays an essential role in viral attachment, fusion, entry, and transmission. S protein comprises an N-terminal S1 subunit responsible for virus-receptor binding and a C terminal S2 subunit responsible for virus-cell membrane fusion. S1 is further divided into an N-terminal domain (NTD) and a receptor-binding domain (RBD), RBD within S1 directly interacts with host receptors. Human angiotensin coverting enzyme 2 (hACE2) is the receptor to which SARS-CoV-2 binds to enter host cells.
Infection with SARS-CoV-2 initiates an immune response, which includes the production of antibodies, or binding antibodies, in the blood. Not all binding antibodies can block cellular infiltration and replication of the SARS-CoV-2 virus. The subpopulation of the binding antibodies that can block cellular infiltration and replication of the virus are named NAbs. RBD within S1 subunit is the most critical target for SARS-CoV NAbs. Such NAbs can interrupt the interaction of RBD and its receptor ACE2. Thus, SARS-CoV-2 NAbs level in human serum correlates with protective immune responses in individuals who have recovered from SARS-CoV-2 infection and also reflect herd immunity at a population level, informing clinical management of patients with past or ongoing COVID-19 infection. However, it is unknown how long it takes for neutralizing antibodies to be produced, and if they are always produced after SARS-CoV-2 infection.
This test is designed to mimic the virus-host interaction by direct RBD protein-hACE2 protein interaction. The highly specific interaction can then be neutralized, the same manner as in a conventional Virus Neutralization Test (VNT). The kit should not be used to diagnose acute SARS-CoV-2 infection.

TEST PRINCIPLE

Competitive chemiluminescence immunoassay.
The sample, buffer, magnetic microbeads coated with ACE2 antigen and ABEI labeled with recombinant SARS-CoV-2 S-RBD antigen are mixed thoroughly and incubated. SARS-CoV-2 Neutralizing Antibody present in the sample compete with ACE2 antigen immobilized on magnetic microbeads for binding recombinant SARS-CoV-2 S-RBD antigen labeled with ABEI. After precipitation in a magnetic field, decant the supernatant, and perform a wash cycle. Subsequently, the Starter 1+2 are added to initiate a chemiluminescent reaction. The light signal is measured by a photomultiplier as relative light units (RLUs), which is inversely proportional to the concentration of SARS-CoV-2 Neutralizing Antibody presented in the sample.

REAGENTS

Kit Contents

Component	Description	100 tests/kit	50 tests/kit
**Magnetic Microbeads**	Magnetic microbeads coated with recombinant ACE2 antigen, PBS buffer, NaN_3 (<0.09%).	2.5 mL	1.5 mL
**Calibrator Low**	Low concentration of recombinant humanized SARS-CoV-2 Antibody, PBS buffer, NaN_3 (<0.09%).	1.0 mL	1.0 mL
**Calibrator High**	High concentration of recombinant humanized SARS-CoV-2 Antibody, PBS buffer, NaN_3 (<0.09%).	1.0 mL	1.0 mL
**Buffer**	PBS buffer, NaN_3 (0.09%).	6.5 mL	4.0 mL
**ABEI Label**	ABEI labeled with recombinant SARS-CoV-2 S-RBD antigen, PBS buffer, NaN_3 (<0.09%).	7.5 mL	4.5 mL
**Diluent**	PBS buffer, NaN_3 (0.09%).	5.5 mL	3.5 mL
**Control 1**	PBS buffer, NaN_3 (0.09%).	1.0 mL	1.0 mL
**Control 2**	Recombinant humanized SARS-CoV-2 Antibody, PBS buffer, NaN_3 (<0.09%).	1.0 mL	1.0 mL
All reagents are provided ready-to-use.			

Warnings and Precautions

- For *in vitro* diagnostic use.
- For professional use only.
- Exercise the normal precautions required for handling all laboratory reagents.
- Personal protective measures should be taken to prevent any part of the human body from contacting samples, reagents, and controls, and should comply with local operating requirements for the assay.
- A skillful technique and strict adherence to the package insert are necessary to obtain reliable results.
- Do not use kit beyond the expiration date indicated on the label.
- Do not interchange reagent components from different reagents or lots.
- Avoid foam formation in all reagents and sample types (specimens, calibrators and controls).
- All waste associated with biological samples, biological reagents and disposable materials used for the assay should be considered potentially infectious and should be disposed of in accordance with local guidelines.
- This product contains sodium azide. Sodium azide may react with lead or copper plumbing to form highly explosive metal azides. Immediately after disposal, flush with a large volume of water to prevent azide build-up. For additional information, see Safety Data Sheets available for professional user on request.

Note: If any serious incident has occurred in relation to the device, please report to Shenzhen New Industries Biomedical Engineering Co., Ltd. (Snibe) or our authorized representative and the competent authority of the Member State in which you are established.

Reagent Handling

- To avoid contamination, wear clean gloves when operating with a reagent kit and sample. When handling reagent kit, replace the gloves that have been in contact with human specimens, since introduction of sample will result in unreliable results.
- Do not use kit in malfunction conditions; e.g., the kit leaking at the sealing film or elsewhere, obviously turbid or precipitation is found in reagents (except for Magnetic Microbeads) or control value is out of the specified range repeatedly. When kit in malfunction conditions, please contact Snibe or our authorized distributor.
- To avoid evaporation of the liquid in the opened reagent kits in refrigerator, it is recommended that the opened reagent kits to be sealed with reagent seals contained within the packaging. The reagent seals are single use, and if more seals are needed, please contact Snibe or our authorized distributor.
- Over time, residual liquids may dry on the septum surface. These are typically dried salts and have no effect on assay efficacy.
- Use always the same analyzer for an opened reagent integral.
- For magnetic microbeads mixing instructions, refer to the Preparation of the Reagent section of this package insert.
- For further information about the reagent handing during system operation, please refer to Analyzer Operating Instructions.

Storage and Stability

- Do not freeze the integral reagents.

- Store the reagent kit upright to ensure complete availability of the magnetic microbeads.
- Protect from direct sunlight.

Stability of the Reagents	
Unopened at 2-8°C	until the stated expiration date
Opened at 2-8°C	6 weeks
On-board	4 weeks

Stability of Controls	
Unopened at 2-8°C	until the stated expiration date
Opened at 2-8°C	6 weeks
Frozen at -20°C	3 months
Frozen and thawed cycles	no more than 2 times

SPECIMEN COLLECTION AND PREPARATION

Specimen Types

Only the specimens listed below were tested and found acceptable.

Specimen Types	Collection Tubes
**Serum**	Tubes without additive/accessory, or tubes containing clot activator or clot activator with gel.
**Plasma**	K2-EDTA

- The sample types listed were tested with a selection of sample collection tubes that were commercially available at the time of testing, i.e. not all available tubes of all manufacturers were tested. Sample collection systems from various manufacturers may contain differing materials which could affect the test results in some cases. Follow tube manufacturers' instructions carefully when using collection tubes.

Specimen Conditions

- Do not use grossly hemolyzed/hyperlipidaemia specimens and specimens with obvious microbial contamination.
- Ensure that complete clot formation in serum specimens has taken place prior to centrifugation. Some serum specimens, especially those from patients receiving anticoagulant or thrombolytic therapy, may exhibit increased clotting time. If the serum specimen is centrifuged before a complete clotting, the presence of fibrin may cause erroneous results.
- To prevent cross contamination, use of disposable pipettes or pipette tips is recommended.

Preparation for Analysis

- Inspect all specimens for foam. Remove foam with an applicator stick before analysis. Use a new applicator stick for each specimen to prevent cross contamination.
- Frozen specimens must be completely thawed before mixing. Mix thawed specimens thoroughly by low speed vortexing or by gently inverting. Visually inspect the specimens. If layering or stratification is observed, mix until specimens are visibly homogeneous. If specimens are not mixed thoroughly, inconsistent results may be obtained.
- Samples should be free of fibrin, red blood cells, or other particulate matter. Such samples may give reliable results and must be centrifuged at 1,500×g for 10 minutes prior to testing. Transfer clarified specimen to a sample cup or secondary tube for testing. For centrifuged specimens with a lipid layer, transfer only the clarified specimen and not the lipemic material.
- The sample volume required for a single determination of this assay is 40 µL.

Specimen Storage

Specimens removed from the separator, red blood cells or clot may be stored up to 3 days at 2-8°C, or 3 months frozen at -20°C. Frozen specimens subjected to up to 3 freeze/thaw cycles have been evaluated.

Specimen Shipping

- Package and label specimens in compliance with applicable local regulations covering the transport of clinical specimens and infectious substances.
- Do not exceed the storage limitations listed above.

Specimen Dilution

- Samples, SARS-CoV-2 Neutralizing Antibody concentrations above the analytical measuring interval, can be diluted with Diluent either automated dilution protocol or manual dilution procedure. The recommended dilution ratio is 1:9. The concentration of the diluted sample must be >3 µg/mL.
- For manual dilution, multiply the result by the dilution factor. For dilution by the analyzers, the analyzer software automatically takes the dilution into account when calculating the sample concentration.

PROCEDURE

Materials Provided

SARS-CoV-2 Neutralizing Antibody (CLIA) assay, control barcode labels.

Materials Required (But Not Provided)

- General laboratory equipment.
- Fully-auto chemiluminescence immunoassay analyzer Maglumi 600, Maglumi 800, Maglumi 1000, Maglumi 2000, Maglumi 2000 Plus, Maglumi 4000, Maglumi 4000 Plus, MAGLUMI X3, MAGLUMI X8.
- Additional accessories of test required for the above analyzers include Reaction Module, Starter 1+2, Wash Concentrate, Light Check, Tip, and Reaction Cup. Specific accessories and accessories' specification for each model refer to corresponding Analyzer Operating Instructions.
- Please use accessories specified by Snibe to ensure the reliability of the test results.

Assay Procedure

Preparation of the Reagent

- Take the reagent kit out of the box and visually inspect the integral vials for leaking at the sealing film or elsewhere. If there is no leakage, please tear off the sealing film carefully.
- Open the reagent area door; hold the reagent handle to get the RFID label close to the RFID reader (for about 2s); the buzzer will beep; one beep sound indicates successful sensing.
- Keeping the reagent straight insert to the bottom along the blank reagent track.
- Observe whether the reagent information is displayed successfully in the software interface, otherwise repeat the above two steps.
- Resuspension of the magnetic microbeads takes place automatically when the kit is loaded successfully, ensuring the magnetic microbeads are totally resuspended homogenous prior to use.

Assay Calibration

- Select the assay to be calibrated and execute calibration operation in reagent area interface. For specific information on ordering calibrations, refer to the calibration section of Analyzer Operating Instructions.
- Execute recalibration according to the calibration interval required in this package insert.

Quality Control

- When new lot used, register the quality control information including target value, range, and lot, etc.
- Scan the control barcode, choose corresponding quality control information and execute testing. For specific information on ordering quality controls, refer to the quality control section of the Analyzer Operating Instructions.

Sample Testing

- After successfully loading the sample, select the sample in interface and edit the assay for the sample to be tested and execute testing. For specific information on ordering patient specimens, refer to the sample ordering section of the Analyzer Operating Instructions.

To ensure proper test performance, strictly adhere to Analyzer Operating Instructions.

Calibration

Traceability: This method has been standardized against the Snibe internal reference standard.

Test of assay specific calibrators allows the detected relative light unit (RLU) values to adjust the master curve.

Recalibration is recommended as follows:
- Whenever a new lot of Reagent or Starter 1+2 is used.
- Every two weeks.
- The analyzer has been serviced.
- Control values lie outside the specified range.
- Each time a new kit is used.

Quality Control

Controls are recommended for the determination of quality control requirements for this assay and should be run in singlicate to monitor the assay performance. Refer to published guidelines for general quality control recommendations, for example Clinical and Laboratory Standards Institute (CLSI) Guideline C24 or other published guidelines[1].

Quality control is recommended once per day of use, or in accordance with local regulations or accreditation requirements and your laboratory's quality control procedures, quality control could be performed by running the SARS-CoV-2 Neutralizing Antibody assay:
- Whenever the kit is calibrated,
- Whenever a new lot of Starter 1+2 or Wash Concentrate is used.

Controls are only applicable with MAGLUMI system and only used matching with the same top seven LOT numbers of corresponding reagents. For each target value and range refer to the label.

The performance of other controls should be evaluated for compatibility with this assay before they are used. Appropriate value ranges should be established for all quality control materials used.

Control values must lie within the specified range, whenever one of the controls lies outside the specified range, calibration should be repeated and controls retested. If control values lie repeatedly outside the predefined ranges after successful calibration, patient results must not be reported and take the following actions:
- Verify that the materials are not expired.
- Verify that required maintenance was performed.
- Verify that the assay was performed according to the package insert.
- If necessary, contact Snibe or our authorized distributors for assistance.

RESULTS

Calculation

The analyzer automatically calculates the concentration in each sample by means of a calibration curve which is generated by a 2-point calibration master curve procedure. The results are expressed in μg/mL. For further information please refer to the operating instructions of MAGLUMI series Fully-auto chemiluminescence immunoassay analyzer.

Interpretation of Results

A study performed with the SARS-CoV-2 Neutralizing Antibody assay was obtained by testing 381 individuals neither SARS-CoV-2 infection nor vaccination, 99% values were ≤ 0.050 μg/mL.

A study performed with the SARS-CoV-2 Neutralizing Antibody assay was obtained by testing 90 individuals confirmed SARS-CoV-2 VNT_{50} ≥ 20, all values were ≥ 0.300 μg/mL. According to the study, 0.300 μg/mL is considered to be an appropriate cut-off for judging the sample VNT_{50} ≥ 20, and the result ≥0.300 μg/mL is considered to be Reactive.

VNT_{50}: virus neutralization titre, defined as the reciprocal value of the sample dilution that showed a 50 % protection of virus growth.
- For samples with concentration near the cut-off, follow-up tests should be performed.
- Results may differ between laboratories due to variations in population. It is recommended that each laboratory establish its own expected ranges.

LIMITATIONS

- This test is suitable only for investigating single samples, not for pooled samples.
- Bacterial contamination of the specimens may affect the test results.
- Assay results should not be used to diagnose or exclude acute SARS-COV-2 infection or to inform infection status.
- It is unknown at this time if the presence of antibodies to SARS-CoV-2 confers immunity to reinfection.
- Results should be used in conjunction with patient's medical history, clinical examination and other findings.
- If the SARS-CoV-2 Neutralizing Antibody results are inconsistent with clinical evidence, additional testing is needed to confirm the result.
- Heterophilic antibodies in human serum can react with reagent immunoglobulins, interfering with *in vitro* immunoassays. Patients routinely exposed to animals or animal serum products can be prone to this interference and anomalous values may be observed[2].

SPECIFIC PERFORMANCE CHARACTERISTICS

Representative performance data are provided in this section. Results obtained in individual laboratories may vary.

Precision

Precision was determined using the assay, samples and controls in a protocol (EP05-A3) of the CLSI (Clinical and Laboratory Standards Institute): 3 runs per day in duplicate for 5 days at 3 sites (n = 90). The following results were obtained:

Sample	Mean (μg/mL)	N	Repeatability		Between-Lot		Between-Day		Between-Site		Reproducibility	
			SD (μg/mL)	%CV	SD (μg/mL)	%CV	SD (μg/mL)	%CV	SD (μg/mL)	%CV	SD (μg/mL)	%CV
S1	0.079	90	0.002	2.53	0.001	1.27	0.001	1.27	0.004	5.06	0.005	6.33
S2	0.347	90	0.007	2.02	0.002	0.58	0.005	1.44	0.015	4.32	0.017	4.90
S3	1.487	90	0.032	2.15	0.005	0.34	0.008	0.54	0.061	4.10	0.070	4.71
S4	7.069	90	0.145	2.05	0.023	0.33	0.082	1.16	0.345	4.88	0.383	5.42
S5	21.192	90	0.446	2.10	0.060	0.28	0.214	1.01	1.338	6.31	1.428	6.74
QC1	<0.040	90	-	-	-	-	-	-	-	-	-	-
QC2	0.484	90	0.010	2.07	0.002	0.41	0.003	0.62	0.025	5.17	0.027	5.58

Linear Range

0.050-30 μg/mL (defined by the Limit of Quantitation and the maximum of the master curve).

Reportable Interval

0.045-300 μg/mL (defined by the Limit of Detection and the maximum of the master curve×Recommended Dilution Ratio).

Analytical Sensitivity

Limit of Blank (LoB) =0.030 μg/mL.
Limit of Detection (LoD) =0.045 μg/mL.
Limit of Quantitation (LoQ) =0.050 μg/mL.

Interference

Interference was determined using the assay, three samples containing different concentrations of analyte were spiked with potential endogenous and exogenous interferents in a protocol (EP7-A2) of the CLSI. The measurement deviation of the interference substance is within ±10%. The following results were obtained:

Interference	No interference up to	Interference	No interference up to	Interference	No interference up to
Bilirubin	40 mg/dL	Fluticasone propionate	2.5 mg/dL	Ceftriaxone sodium	81.03 mg/dL
Triglycerides	1000 mg/dL	Levofloxacin	1.776 mg/dL	Mometasone	2.5 mg/dL
Hemoglobin	2000 mg/dL	Azithromycin	1.201 mg/dL	Budesonide	3.2 mg/dL
HAMA	30 ng/mL	Ribavirin	90 mg/dL	Mucin	260 mg/dL

RF	1500 IU/mL	Meropenem	80.15 mg/dL	Zanamivir	1.2 mg/dL
Albumin	6 g/dL	Tobramycin	2.4 mg/dL	Peramivir	60 mg/dL
Anti-Mitochondrial	1:64 (titer)	Oseltamivir	1.0 mg/dL	Lopinavir	48 mg/dL
Total IgG	1600 mg/dL	Oxymetazoline	2.5 mg/dL	Ritonavir	120 mg/dL
Total IgM	280 mg/dL	Sodium chloride	45 mg/dL	Arbidol	36 mg/dL
Total IgA	500 mg/dL	Beclomethasone	2.5 mg/dL	Flunisolide	2.5 mg/dL
Interferon α	1500 U/mL	Dexamethasone	18 mg/dL	Histamine dihydrochloride	4.5 mg/dL
Phenylephrine hydrochloride	1.0 mg/dL	Triamcinolone acetonide	5.5 mg/dL	Biotin	5.0 mg/dL

Cross-Reactivity
The cross-reactivity study for the SARS-CoV-2 Neutralizing Antibody assay was designed to evaluate potential cross reactants. The results are listed in the following table:

Category	N of samples	Reactive	Category	N of samples	Reactive
Human Coronavirus antibodies (HKU1, OC43, NL63, 229E)	20	0	Human immunodeficiency virus antibodies	12	0
Influenza A virus antibodies	18	0	Hepatitis C virus antibodies	10	0
Influenza B virus antibodies	13	0	Hepatitis B virus antibodies	12	0
Respiratory syncytial virus antibodies	7	0	M.Pneumonia antibodies	7	0
CMV antibodies	10	0	Adenovirus antibodies	10	0
EB virus antibodies	15	0	ANA	6	0

Clinical Sensitivity
The clinical sensitivity of the SARS-CoV-2 Neutralizing Antibody assay was determined by testing 57 samples confirmed SARS-CoV-2 $VNT_{50} \geq 20$.

N of samples	Reactive	Sensitivity	95% CI
57	57	100%	93.69%-100.00%

Clinical Specificity
The clinical specificity of the SARS-CoV-2 Neutralizing Antibody assay was determined by testing 120 samples from subjects neither SARS-CoV-2 infection nor vaccination.

N of samples	Non-reactive	Specificity	95% CI
120	120	100.0%	96.90%-100.00%

REFERENCES
1. CLSI. Statistical Quality Control for Quantitative Measurement Procedures: Principles and Definitions. 4th ed. CLSI guideline C24. Wayne, PA: Clinical and Laboratory Standards Institute; 2016.
2. Boscato L M, Stuart M C. Heterophilic antibodies: a problem for all immunoassays [J]. Clinical Chemistry, 1988,34(1):27-33.

Shenzhen New Industries Biomedical Engineering Co., Ltd.
No.23, Jinxiu East Road, Pingshan District, 518122 Shenzhen, P.R. China
Tel: +86-755-21536601 Fax: +86-755-28292740

EC REP
Shanghai International Holding Corp. GmbH (Europe)
Eiffestrasse 80, 20537 Hamburg, Germany
Tel: +49-40-2513175 Fax: +49-40-255726

SYMBOLS EXPLANATIONS

	Consult instructions for use		Manufacturer
2 °C 8 °C	Temperature limit (Store at 2-8°C)		Use-by date
Σ	Contains sufficient for <n> tests		Keep away from sunlight
	This way up	EC REP	Authorized representative in the European Community
IVD	*In vitro* diagnostic medical device	CONTENTS	Kit components
REF	Catalogue number	LOT	Batch code
CE	CE marking		

Snibe Diagnostic	# Reagent Note	International Technical Service Department

Assay	MAGLUMI® SARS-CoV-2 S-RBD IgG (CLIA)<br>MAGLUMI® SARS-CoV-2 Neutralizing Antibody (CLIA)		
Subject	WHO Standard (20/136) Unit Conversion		
REF No.	RN21040201	***Issued Date***	April 2nd, 2021

**Immediate action required**	
**Recommendation**	
**For your information**	√

Dear customers

We have the calibrated the SARS-CoV-2 S-RBD and SARS-CoV-2 Neutralizing antibody kits with the WHO standard (20/136).

By analyzing and calculating the test results of WHO standard (20/136), the following unit conversion relationships are obtained.

1.For SARS-CoV-2 S-RBD IgG reagent, 1 AU/mL is equivalent to 4.33 BAU/mL.

2.For SARS-CoV-2 Neutralizing Antibody reagent, 1 μg/mL is equivalent to 405 IU/mL. (It is based on the conversion of 0.3 μg/mL equivalent to 121.5 IU/mL with the SARS-CoV-2 Neutralizing Antibody assay by testing 90 individuals confirmed SARS-CoV-2 $VNT_{50} \geqslant 20$, all values were $\geqslant$ 0.300 μg/mL.)

Snibe International Technical Service Department
