# Supplementary material for: Cellular and Humoral Responses to Recombinant and Inactivated SARS-CoV-2 Vaccines in CKD Patients: An Observational Study
Source: J Clin Med. 2023 Feb 3;12(3):1225. doi: 10.3390/jcm12031225 (PMC9918183; doi:10.3390/jcm12031225)
Supplement: Supplementary file 1 [file jcm-12-01225-s001.zip › Supplementary Material S3.pdf]

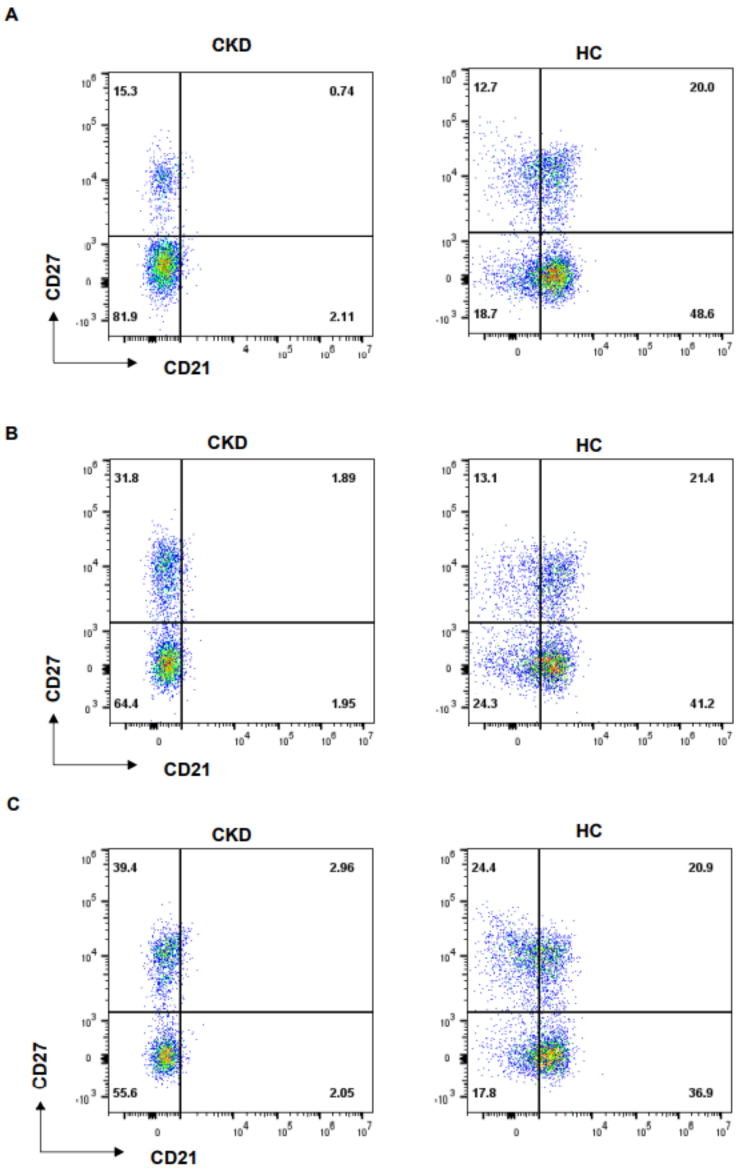


Examples of flow plots of figures. **A:** Examples of flow plots of figure 1E,1F,1G and 1H. **B:** Examples of flow plots of figure 3E,3F,3G and 3H. **C:** Examples of flow plots of figure 4E,4F,4G and 4H.
