# Supplementary material for: Cellular and Humoral Responses to Recombinant and Inactivated SARS-CoV-2 Vaccines in CKD Patients: An Observational Study
Source: J Clin Med. 2023 Feb 3;12(3):1225. doi: 10.3390/jcm12031225 (PMC9918183; doi:10.3390/jcm12031225)
Supplement: Supplementary file 1 [file jcm-12-01225-s001.zip › Supplementary Material S5 Questionnaire on Adverse Reactions to COVID-19 vaccination.pdf]

Questionnaire on Adverse Reactions to COVID-19 vaccination						
Name	Gender	Age	contact information			
Type of COVID-19 vaccine			Group			
adverse reaction	Level of adverse reactions (1-4 for each adverse reaction)					
	The first vaccination		The second vaccination		The third vaccination	
	Within 7 days	Within 30 days	Within 7 days	Within 30 days	Within 7 days	Within 30 days
Local pain (affecting limb movement)						
Local swelling (diameter)						
Local induration (diameter)						
Local redness (diameter)						
Local rash (diameter)						
Local itching (48h)						
Phlegmon						
Fever (37.3, 38, 38.5)						
Cough (treatment or not)						
Difficulty breathing (when)						
Anorexia						
Nausea (duration)						
Vomiting (number of times)						
Constipation (treatment or not)						
Diarrhea (frequency/day, fecal character)						
Myalgia (non-vaccination site, activity)						
Arthralgia (Daily activities)						
Arthritis (pain level, activity)						
Headache (affecting activity)						
Syncope(loss of consciousness or not)						
Insomnia (Affecting life)						
Fatigue (affects activity)						
Mental disorders (anxiety, depression, etc.)						
Acute allergic reaction (urticaria, treatment or not)						
Others						
