# Supplementary figures and images for: Cellular and Humoral Responses to Recombinant and Inactivated SARS-CoV-2 Vaccines in CKD Patients: An Observational Study
Source: J Clin Med. 2023 Feb 3;12(3):1225. doi: 10.3390/jcm12031225 (PMC9918183; doi:10.3390/jcm12031225)

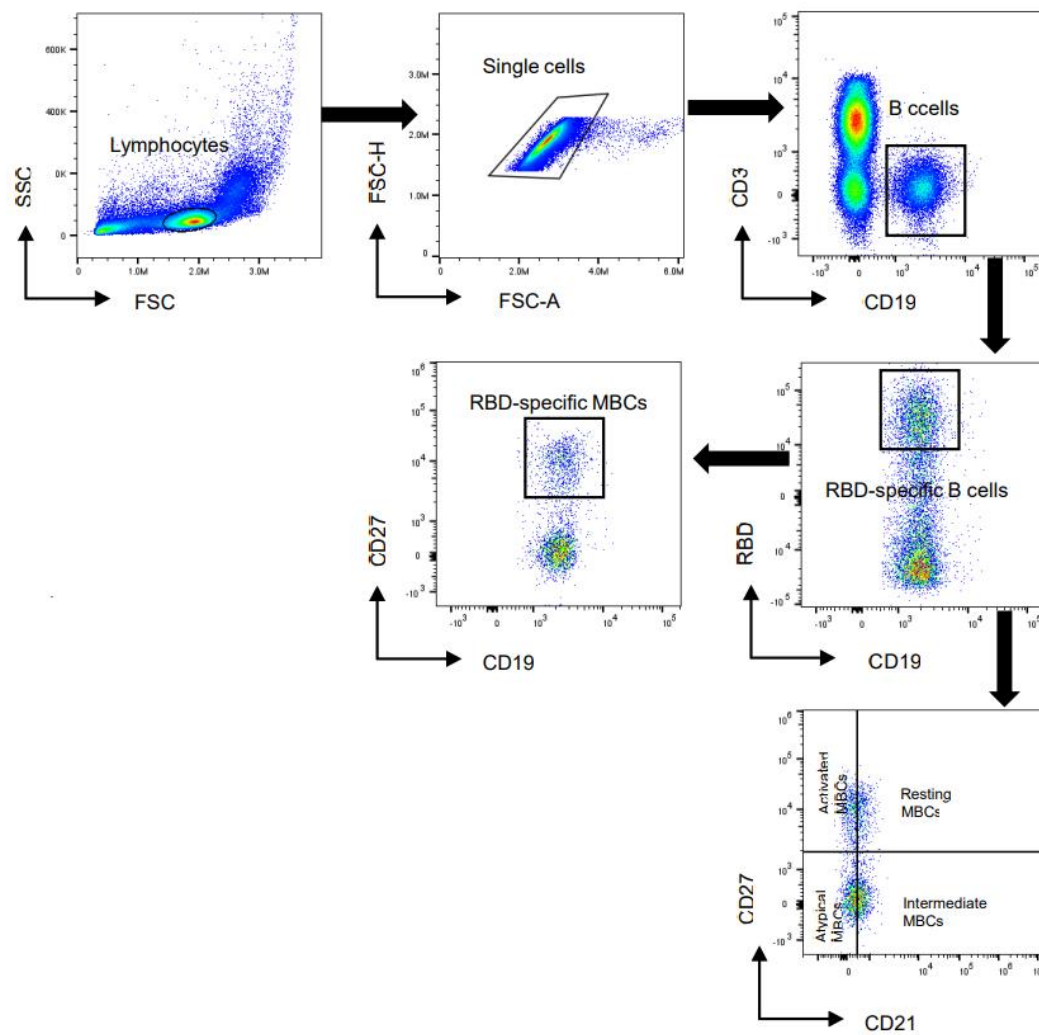

The full gating strategy of flow cytometry for target cell populations.

Supplement: Supplementary file 1 [file jcm-12-01225-s001.zip › Supplementary Material S2.pdf]
